# Cloning capacity helps seeds of *Garcinia xanthochymus* counter animal predation

**DOI:** 10.1002/ece3.8008

**Published:** 2021-08-23

**Authors:** Zhen‐yu Wang, Lin Cao, Chuan Yan, Yu‐da Niu, Kang Chong, Zhi‐bin Zhang

**Affiliations:** ^1^ State Key Laboratory of Integrated Management of Pest Insects and Rodents in Agriculture Institute of Zoology Chinese Academy of Sciences Beijing China; ^2^ Key Laboratory of Poyang Lake Wetland and Watershed Research (Ministry of Education) College of Life Sciences Jiangxi Normal University Nanchang China; ^3^ College of Ecology and Environmental Science Institute of Ecology and Geobotany Yunnan University Kunming China; ^4^ Institute of Botany Chinese Academy of Sciences Beijing China

**Keywords:** cloning strategy, mutualism, rodent, seed dispersal, seed predation, seedling establishment

## Abstract

Seed predators have the potential to act as agents of natural selection that influence seed traits and seed fates, which in turn affect the whole plant population dynamic. Accordingly, plants deploy a variety of mechanisms (e.g., resistance and tolerance strategies) to lessen the impact of predation on seed crop or on an individual seed. In this study, we described a novel mechanism, seed cloning strategy, in a tropical plant species in countering animal predation. By conducting field‐ and laboratory‐based germination experiments, we found that both rodent damaged and artificially damaged seed fragments of a large‐seeded tree *Garcinia xanthochymus* (Clusiaceae) could successfully germinate and establish as seedlings. Tissue culture experiments revealed that *G. xanthochymus* has no endosperm in seeds, and its seed fragments own strong capacity of differentiation and cloning. Seed damage negatively affected seedling growth and germination, but the seed germination rate was remarkably high. Our study suggests that, seed cloning capacity, adopted by the large‐seeded tree *G. xanthochymus* may act as a novel strategy counteract for seed predation and would play a significant role in stabilizing the mutualism between plant and animals.

## Graphical abstract

We revealed a distinct evolutionary strategy of seed cloning in countering animal predation, as compared to previous ones such as tolerance and resistance.

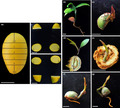

## 1 INTRODUCTION

Seed dispersal and seed predation are important ecological processes which affecting plant recruitment, spatial distribution, and species coexistence (Jansen et al., 2014; Wang et al., 2019). Many plants with large‐sized seeds rely heavily on animals for seed dispersal (Lichti et al., 2017; Vander Wall, 2001). Plants produce a large amount of nutrient‐rich fruits or seeds for attracting animals to disperse and compensate for predispersal seed losses to seed predators (Bruno et al., 2020; Corlett, 2017). Animals consume these fruits or seeds to obtain their required energy or to remove and store seeds away from their parent trees for future use (Hirsch et al., 2012; Hollander & Vander Wall, 2004). Seed consumption by animals would result in a predatory interaction with trees, while seed dispersal would result in a mutualistic if dispersed seeds escape from predation by animals and establish into seedlings (Briggs et al., 2009; Gómez et al., 2019). Because animal predation on plant seeds is often very high (Blendinger & Diaz‐Velez, 2010; Hulme & Hunt, 1999), seed predation may impose strong selection pressure on evolution of seed traits (Cao et al., 2011; Janzen, 1971). Thus, plants may have evolved a variety of mechanisms to counter over predation by animals.

Plants are known to adopt resistance (Chen et al., 2012; Grubb et al., 1998; Zhang & Zhang, 2008) and tolerance mechanisms (Dalling & Harms, 1999; Perea et al., 2011; Vallejo‐Marin et al., 2006; Xiao et al., 2007) for countering the seed predation by animals. Resistance mechanisms involve physical traits (e.g., thick exocarp, spiny fruits, hard seed coats) or chemical traits (toxic and unpalatable chemical compounds) that reduce consumption rate of plant seeds and/or negatively affect predator's performance (Guimaraes et al., 2003; Shimada et al., 2015; Strauss & Agrawal, 1999; Zhang et al., 2016). However, excessively high defensive traits of seeds may reduce attractiveness and therefore dispersal effectiveness by animals (Vander Wall, 2010; Zhang et al., 2016). Thus, plants developed tolerant seed traits to increase seed escape and consequential survival under extensive animal predation (Loayza et al., 2014; Xiao et al., 2007). Tolerance mechanisms mean that the seed is able to germinate and develop into a seedling after being partially eaten or damaged by animals, and however, sometimes it develops as a seedling with lower fitness than intact seed (Dalling & Harms, 1999; Vallejo‐Marin et al., 2006). Seeds with strong tolerance to animal predation are often large and contain a nutrient‐rich endosperm or cotyledons, which make it possible for partially damaged seeds to successfully germinate and establish into seedlings (Mendoza & Dirzo, 2009; Perea et al., 2018).

The embryo in a seed is essential for seed germination and seedling establishment, but it can be easily destroyed by seed consuming animals. In nut‐bearing trees, particularly in oak (genus *Quercus*) acorns, embryo damage is common and leads, in general, to seed death (Branco et al., 2002; Perea et al., 2011; Steele et al., 2001; Xiao et al., 2009; Yang et al., 2012), resulting in a transition from mutualism to predation. However, a few studies have documented that survival and germination of embryo‐damaged acorns can occur if the vulnerable embryo is partially retained, but with significantly lower germination rates as compared to intact acorns (Bartlow et al., 2018; McEuen & Steele, 2005; Xiao et al., 2010; Yi & Yang, 2010). Seed with proportionally larger embryos (radicle plus plumule) provide greater tolerance to seed damage by rodents, allowing successful germination (Perea et al., 2018). A few studies reported that some plant species had seed fragments that were able to establish into seedlings after damage by rodents or artificially damaged by people (Cao et al., 2011; Joshi et al., 2006; Teixeira & Barbedo, 2012), suggesting that seeds may possess regeneration capacity in order to counter for animal predation.

Cloning is a common phenomenon in plants as an asexual reproduction strategy. In many plant species, tissues of leaves, roots, or stems can easily develop into seedlings (Thorpe, 2007). However, the cloning capacity of seed fragments and its link to animal predation has been quantified in a few previous studies (Cao et al., 2011; Joshi et al., 2006; Teixeira & Barbedo, 2012), but it is rarely evaluated in field conditions under animal predation. In the Xishuangbanna tropical forests in Yunnan, China, *Garcinia xanthochymus* is an common evergreen tree that produces a sugary water‐rich pulp which attract frugivorous vertebrates, such as elephants, deer, and primates for seed dispersal (Kitamura et al., 2002; Stevenson et al., 2000). Due to extensive human disturbances in the Xishuangbanna region during past decades, populations of large vertebrates have significantly declined, or even suffered local extinction (Cao et al., 2011). By using infrared cameras, Wang et al., 2019 found there were very few large vertebrates (*Macaca mulatta*, *Paguma larvata taivana*) visiting the fruiting trees; piles of *G. xanthochymus* fruit were rotten under parent trees. Many small rodents (e.g., *Maxomys surifer* and *Niviventer confucianus*) were found frequently eating and removing the *G. xanthochymus* seeds. The rate of seedling recruitment was over 20%, two times larger than that of the sympatric tree *Scleropyrum wallichianum*, which indicated that scatter‐hoarding rodents play a significant role in seed dispersal and seedling recruitment of *G. xanthochymus* (Wang et al., 2019). In addition, almost 15% seeds of *G*. *xanthochymus* were only partially eaten by rodents although these seeds have very thin aril coats, many seed fragments left on the ground after rodent predation germinated and developed into seedlings (Unpublished data). The emergence of clonal seedlings from any seed fragments may be used in countering animal predation similar with other Garcinia species (Anto et al., 2018; Joshi et al., 2006; Malik et al., 2005), but field tests on animal or artificial damaged seed fragments have not been conducted. In this study, by conducting field‐ and laboratory‐based germination experiments with rodent damaged and artificially damaged seeds, we want to verify whether seeds of *G. xanthochymus* possessed the cloning capacity to account for rodent predation. We also conducted in vitro tissue culture experiments to examine the cloning capacity of *G. xanthochymus* seeds.

## 2 MATERIALS AND METHODS

### 2.1 Study site and species

We conducted this study in the Menglun Nature Reserve, Xishuangbanna, Yunnan, China. This region is dominated by a typical tropical monsoon climate with distinct rainy (May to October) and dry seasons (November to April). The average annual temperature of this area is 21.8°C. The annual precipitation varies from 1,200 to 1,700 mm, of which approximately 85% occurs during the rainy season. The dry season is characterized by heavy radiation fog, which supplements the deficiency of rainfall in this season (Zhang & Cao, 1995).

**FIGURE 1:**
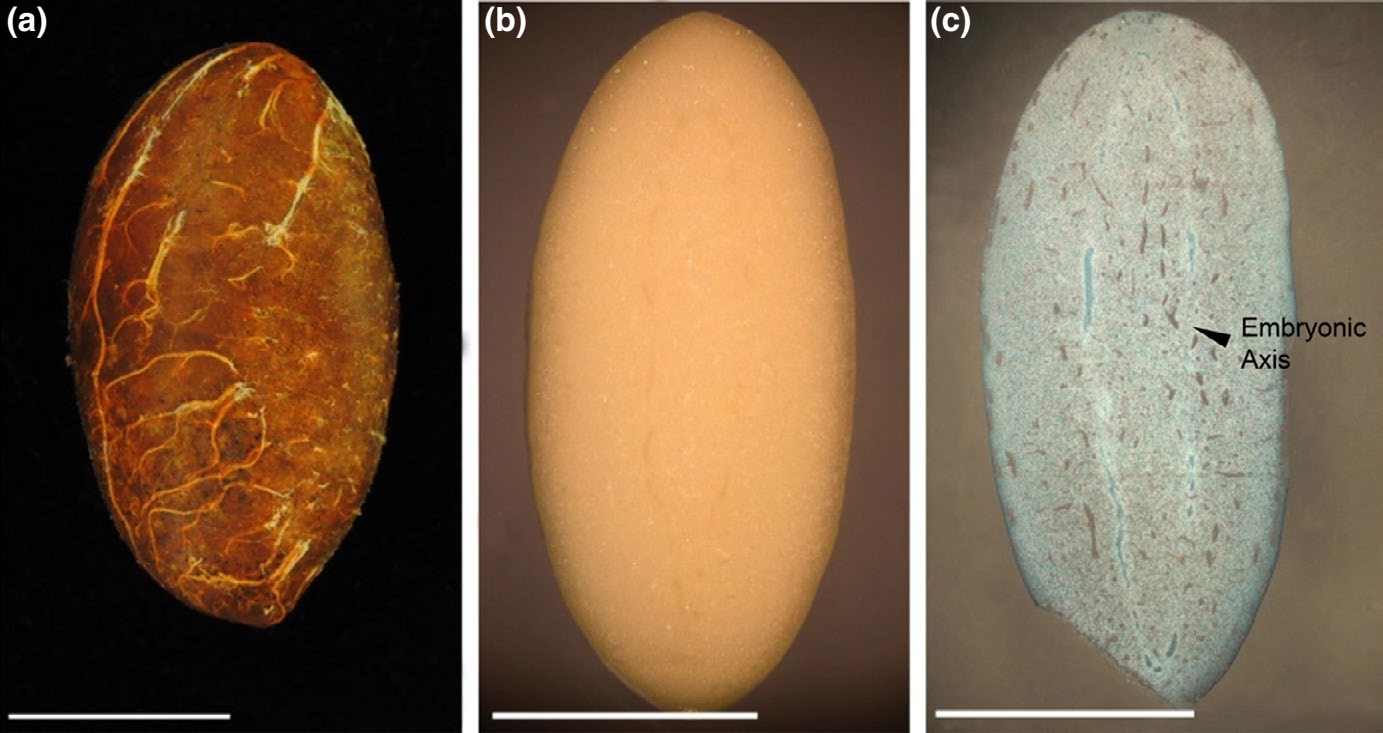
The anatomical diagram of *G. xanthochymus* seed. (a) Intact seed; (b) Longitudinal section of seeds; (c) Longitudinal paraffin section of seeds

*G. xanthochymus* of the Clusiaceae family is a common evergreen tree that is widely distributed in the Xishuangbanna tropical forests in southwest China. Its flowering period is from March to May, and ripe fruit is available from September to November. Fruit crop per individual tree is highly variable, ranging from 31 to 357 fruits (average 256 ± 143, mean ± *SD*, *n* = 8). Ripe fruits are globose or ovoid, fleshy, yellow berries that generally contain 1–3 arillate seeds. The fruit mass is 118.04 ± 26.15 g, fruit length is 6.21 ± 0.64 cm, and fruit diameter is 6.01 ± 0.56 cm (*n* = 60). Seeds are oblong or ovoid, seed mass is 4.35 ± 0.9 g, seed length is 3.01 ± 0.75 cm, and seed diameter is 1.67 ± 0.25 cm (*n* = 60). Seeds have a thin testa (<1 mm) and low tannin (1.56%) and also rich in nutrients (up to 28.26% fat content, 38.2% starch content). Seeds are reported to show dormancy owing to an impermeable seed testa similar with other Garcinia species (Anto et al., 2018; Malik et al., 2005). There are no differentiated embryos, endosperm, or embryonic axis in seeds of *G. xanthochymus* similar with other Garcinia species (Figure 1; Malik et al., 2005; Noor et al., 2016).

### 2.2 Seed collection

Seeds were obtained from mature *G. xanthochymus* fruits in October 2010 from eight individuals. All fruits were subsequently taken to the laboratory, where we manually removed seeds from the fruits, without damaging the seed coat. We used only intact seeds for our experiments. During seed collection process and our previous field study (Wang et al., 2019), we found no insects eat seeds of *G. xanthochymus*.

### 2.3 Seed predation and hoarding by rodents

We studied seed predation and hoarding behavior of two dominant rodent species, red spiny rats *M. surifer* and Chinese white‐bellied rats *N. confucianus* on seeds of *G. xanthochymus* by using four 10 × 10 m semi‐natural enclosures (for details of the enclosure see: Wang et al., 2014). To prevent rodents escaping from the enclosures and to prevent other animals from entering it, the walls of the enclosures were built using concrete and poured 0.5 m below the ground surface level. The top of the enclosures was covered with a plastic sheet to prevent rain from entering during the experiments. One underground nest was provided for the rodents in the corner of each enclosure. We captured rodents in the field using live traps (L × W × H = 30 × 14 × 14 cm) made of steel wire mesh. Each trap was baited with fresh peanuts and checked before sunrise and sunset. The species name, body weight, and reproductive status of each captured animal were recorded before bringing them to the indoor. Pregnant females or juveniles were immediately released in the field in situ. Ten healthy adult *N. confucianus* (4 females and 6 males, average body weight 85.4 ± 9.7 g) and sixteen *M. surifer* (9 females and 7 males, average body weight 117.9 ± 10.9 g) were used in this experiment. All adult animals were kept individually in a cage (L × W × H = 40 × 25 × 30 cm) and provided with adequate food, water, and nest material. A photoperiod cycle of 12:12 hr (light:dark) was maintained. During the experiments, one animal was placed in the enclosure and observed for two consecutive days. The animal was provided with laboratory food on the first day in order to ease adaptation to the new environment. On the second day, twenty intact seeds of *G. xanthochymus* were placed in the center of enclosure. Seeds were marked by attaching a small coded plastic tag to each seed through a thin steel thread (for details see: Wang et al., 2014). Seeds were classified as eaten seeds, scatter‐hoarded seeds, or larder‐hoarded seeds by following Wang et al.  2018 seed fragments of *G. xanthochymus* after being partially eaten by rodents in this experiment were collected and used for the following field germination experiments. This study followed the ASAB/ABS Guidelines for the care and use of laboratory animals of China and was approved by the Institute of Zoology, Chinese Academy of Sciences.

### 2.4 Germination experiments of rodent and artificially damaged seed fragments

**FIGURE 2:**
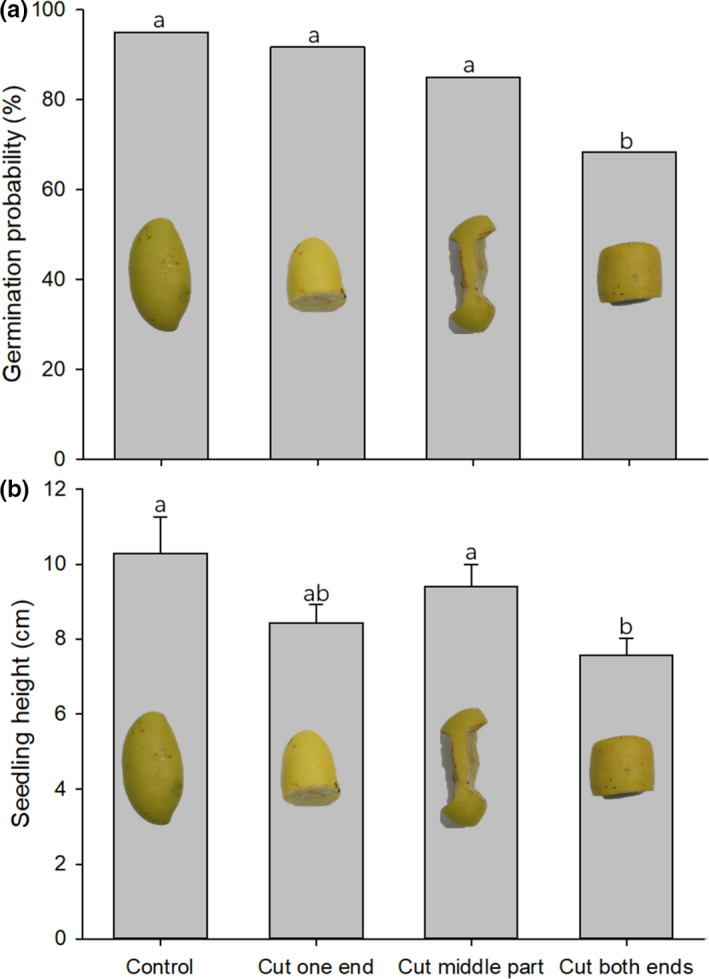
The germination rates (a, mean ± *SE*) and the average seedling height (b, mean ± *SE*) of seeds of *G. xanthochymus* under the different treatments. Different letters indicate statistically significant at *P* < .05 level

In October 2010, we set up a small enclosure for germination experiments in the gully rain forest in the Xishuangbanna Tropical Botanical Garden of the Chinese Academy of Sciences. The enclosure (L × W × H = 3 × 1.2 × 0.6 m) was surrounded and covered by wire mesh (mesh size = 1 × 1 cm) and extended 20 cm into the soil to prevent from entry of vertebrates, especially rodents. The enclosure was divided into small 10 × 10 cm grids by using nylon ropes, one grid square was used for one seed. To study the consequences of partial seed predation on the germination and seedling survival of *G. xanthochymus* seeds, three artificial cutting treatment groups were conducted: (a) removal of one end of the intact seed; (b) removal of both ends of the intact seed; and (c) substantial removal of the middle part of the intact seed (Figure 2). Each treatment consisted of 60 artificially damaged seed fragments. To evaluate the effect of the seed fragment size on germination rate and growth, each treatment group was divided into three subgroups, with the proportion of removed part being controlled into three categories: <25%, 25%–50%, and >50%. The sample size of each subgroup was 20 seeds. Each seed was individually weighed before and after cutting to calculate the mass removed by artificial cutting. And within each subgroup, the proportion of removed mass was randomly distributed. In addition to the artificial cutting treatment, a control group consisting of 20 intact seeds and a rodent predation group consisting partially eaten seeds by rodents (here defined as the rodent damaged seed fragments, *n* = 42) that were collected in the enclosure experiments were also evaluated. In November 2010, all tested seeds were sown individually and randomly in the small grids except the grids around the edge of the enclosure. A coded plastic tag (2.5 × 3.6 cm) was attached to the nylon ropes of the small grids to distinguish the seeds of each treatment group. In order to simulate the scatter‐hoarding behavior of rodents, we buried the tested seeds in the shallow surface and covered them with a layer of fallen leaves. The dormancy period for *G. xanthochymus* seeds is more than four months (Joshi et al., 2006). From the end of March 2011, seeds were monitored for germination and sprouting, and after seedling establishment, leaf production and growth in height were measured once a week. We recorded the time of germination and the percentage of germination for intact seeds, artificially damaged seed fragments and rodent damaged seed fragments. A seed or its fragment was defined as germinated when a shoot grew out of the soil and was clearly visible. We also quantified seedling establishment rates when germinated seeds produced a main stem with its first permanent leaf.

### 2.5 Laboratory germination experiments

We removed the testa of *G*. *xanthochymus* seeds to break seed dormancy and planted them in nutritious soil to observe their germination and seedling establishment. The culture room temperature was maintained at 30°C, and the photoperiod cycle was 12:12 hr (light:dark). The seeds were considered as germinated when the length of the primary shoot was ≥1 cm.

### 2.6 Laboratory tissue culture experiments

We studied cloning capacity of artificially damaged seed fragments of *G. xanthochymus* through tissue culture in laboratory. Firstly, we removed the testa of intact seeds and then soaked them in 70% alcohol for 30 s. Secondly, using sterile distilled water, we rinsed the seed twice and then transferred it to 0.1% mercuric chloride solution for 15 min. Thirdly, we rinsed seeds with sterile distilled water five times and then blotted the moisture with sterile filter paper. After cutting seeds into two halves longitudinally, we put them in 1/2 MS (Murashige and Skoog medium) medium for culturing. During the cultivation process, we found that two seedlings could be formed on the top of the longitudinally sliced seed; thus, we cross‐cut the seed and cut both ends longitudinally into eight small pieces (Figure 7a) and placed the pieces on medium supplemented with different cytokinin (BA, 6‐Benzylaminopurine) and auxin (NAA, 1‐Naphthylacetic acid) with matching ratios (Table 1). We evaluated the effects of different mediums on plant regeneration and counted the number of regenerated plants. Eight seeds were used as replicates for each medium. The medium in this experiment included the following four types: (1) 1/2 MS (control groups); (2) 1/2 MS + 0.5 mg/L 6‐BA + 0.25 mg/L NAA; (3) 1/2 MS + 0.5 mg/L 6‐BA; and (4) 1/2 MS +0.25 mg/L NAA (Table 1). We added 15 g/L sucrose and 7 g/L agar into the medium, adjusted PH value to 5.8, and then sterilized them for 20 min at 121°C. A photoperiod cycle of 12:12 hr (light:dark) and temperature 25 ± 2°C was maintained in the culture room. Test materials were subcultured every two weeks. After subculturing for about two months, complete plants were formed.

**TABLE 1:** The number of cloning seedlings of *G. xanthochymus* seed after multiple cuts and culturing in different mediums (BA is 6‐benzylaminoadenine one kind of cytokinin; NAA is 1‐naphthylacetic acid one kind of auxin)

Culture mediums	Seeds (*n*)	Seed fragments (*n*)	Number of cloning seedlings emerging (*n*)
1/2 MS	8	64	16
1/2 MS + 0.5 mg/L 6‐BA	8	64	18
1/2 MS + 0.25 mg/L NAA	8	64	17
1/2 MS + 0.5 mg/L 6‐BA + 0.25 mg/L NAA	8	64	26

### 2.7 Statistical analysis

The difference of germination rate between the control seeds and rodent damaged seed fragments was compared by chi‐squared test. The difference on seed germination rate and seedling height among treatments (control, cut one end, cut middle part, and cut both ends) was compared by generalized linear models (GLMs) with binomial distribution and Gaussian distribution, respectively. We also used GLM with binomial distribution to analyze the effects of seed mass lost (%) or seed mass retained (g) after artificial seed cutting, including its interaction with treatments on seed germination rate in the model. Similarly, GLM with Gaussian distribution was applied to analyze their effects on seedling height. The overall significance of treatments was tested by Wald test. All data were analyzed using R software (R Core Team, 2019), and post hoc Tukey test was performed by Tukey test through “lsmeans” package.

## 3 RESULTS

### 3.1 Seed predation and hoarding by rodents

For each experimental animal, we provide it with 20 intact seeds of *G. xanthochymus*. Sixteen red spiny rats (*M*. *surifer*) harvested 15.9 ± 1.5 (mean ± *SE*) seeds of *G. xanthochymus* within 24 hr, ate 2.3 ± 0.5 seeds, and scatter‐hoarded 13.6 ± 1.4 seeds. Ten Chinese white‐bellied rats (*N*. *confucianus*) harvested 7.3 ± 1.0 seeds of *G. xanthochymus* within 24 hr, ate 2.4 ± 0.3 seeds, and scatter‐hoarded 1.6 ± 0.5 seeds. Red spiny rats feed on 37 seeds, of which 27 (73%) were only partially eaten. White‐bellied rats consumed 24 seeds, of which 15 (62.5%) were only partially eaten. All rodents had no obvious preference for the seed feeding site.

### 3.2 Germination of rodent damaged seed fragments in field conditions

The intact *G*. *xanthochymus* seeds germinated easily under natural environmental conditions, with a germination rate of 95% (19/20), and all germinated seeds or 100% (19/19) established into seedlings. In contrast, the germination rate and seedling establishment rate of rodent damaged seed fragments (collected from the enclosures after rodent predation) was 38.09% (16/42) and 93.75% (15/16), respectively. The difference of germination rate between the control and rodent damaged seed fragments was statistically significant (Wald test, χ^2 ^= 4.52, *df* = 1, *p* < .05).

### 3.3 Germination of artificially damaged seed fragments in field conditions

Artificially cutting seeds into fragments had a significant effect on the seed germination rate of *G. xanthochymus* (Wald test, *χ* ^2^ = 14.349, *df* = 3, *p* = .002). Cutting both ends of intact seeds significantly reduced the germination success of *G. xanthochymus* seeds as compared with the control group (*p* = .04); there was no significant difference of germination rate between the other treatments and the control group (all *p* > .05; Figure 2a).

**FIGURE 3:**
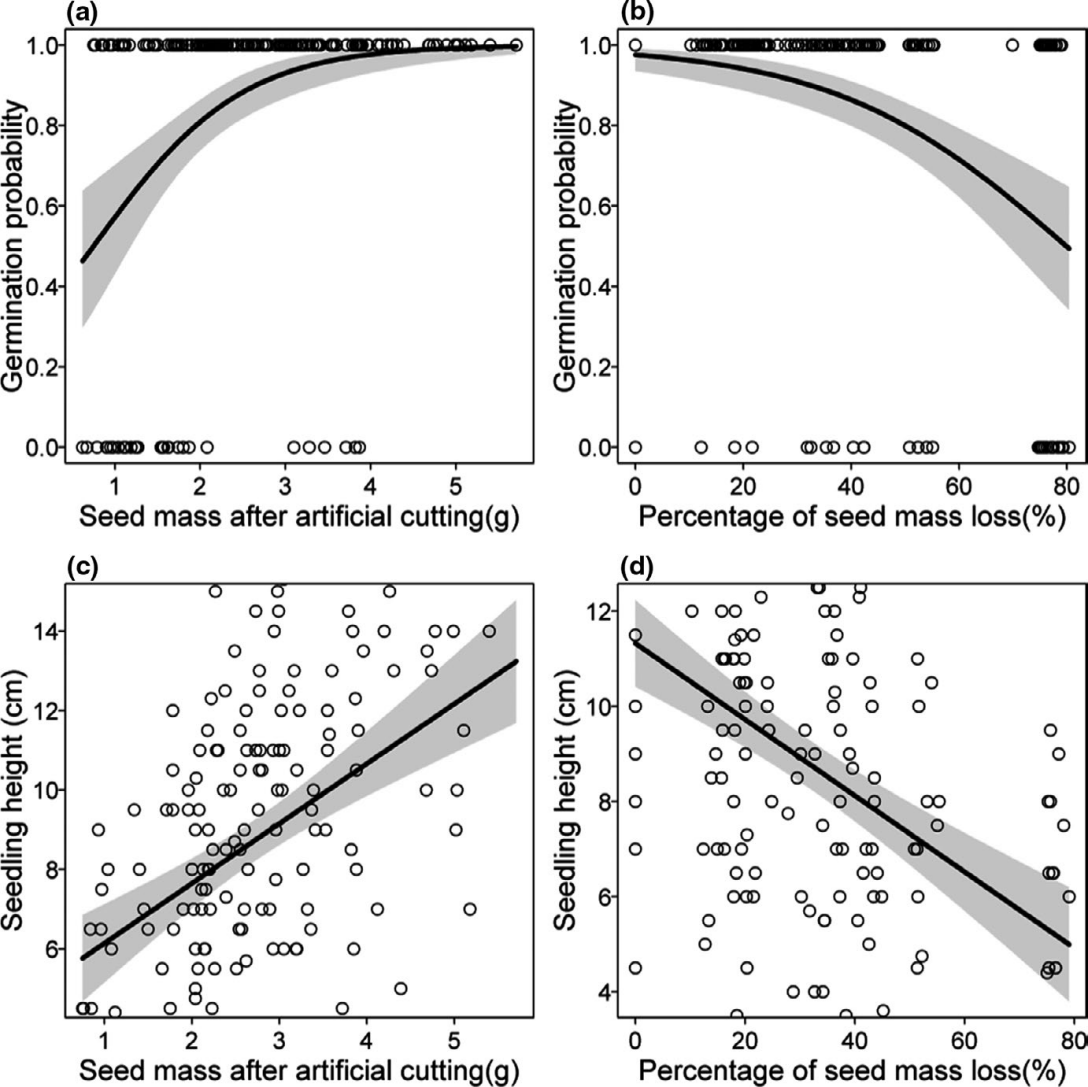
Relationship between germination rate and seed mass retained (a) or percentage of seed mass loss (b) after artificially cutting seeds of *G. xanthochymus*; and relationship between seedling height and seed mass retained (c) or percentage of seed mass loss (d) after artificially cutting seeds of *G. xanthochymus*. The shaded area indicates 95% confidence bands of regression lines

The seed mass after artificial cutting had a significant positive effect on the germination rate (*z* = 4.501, *df* = 199, *p* < .001; Figure 3a). While the percentage of seed mass loss by artificial cutting had a significant negative effect on seed germination rate (*z* = –4.842, *df* = 199, *p* < .001; Figure 3b).

Artificial cutting treatment had a significant effect on *G. xanthochymus* seedling growth (as measured by plant height; *F* = 3.122, *df* = 3, *p* = .028). Cutting both ends of intact *G. xanthochymus* seeds decreased seedling height than the control group (*p* < .05); however, there was no significant difference of seedling height between the other treatments and the control group (all *p* > .05; Figure 2b).

**FIGURE 4:**
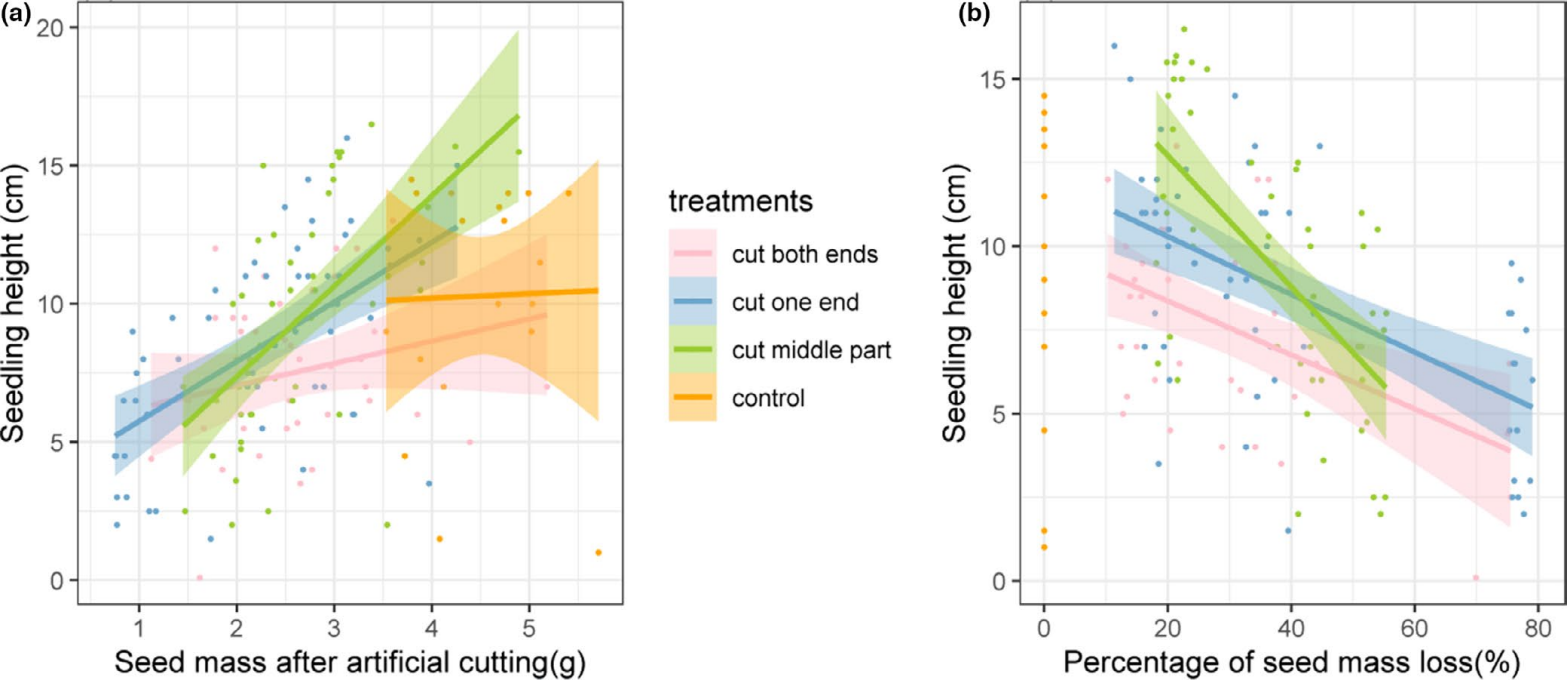
Seed mass retained after artificially cutting is positively associated with seedling height within different treatment groups (a); seed mass loss after artificially cutting is negatively associated with seedling height for different treatment groups (except for control group) (b). The lines indicate the linear regression between seed mass retained and seedling height. The shaded area of each regression indicates 95% confidence bands of regression lines

Seed weight after artificial cutting had a significant positive effect on seedling height. With an increase in seed weight, seedling height increased significantly (*F* = 41.26, *df* = 1, *p* < .001; Figure 3c). There was a significant interaction between artificial cutting treatments and seed mass after artificial cutting on seedling height (*F* = 3.443, *df* = 3, *p* = .018; Figure 4a).

Seed mass loss also negatively affected seedling height (*F* = 57.69, *df* = 1, *p* < .0018; Figure 3d). There was a significant interaction between artificial cutting treatments and the percentage of seed mass loss on seedling height (*F* = 4.6528, *df* = 2, *p* = .011; Figure 4b).

### 3.4 Germination of artificially damaged seed fragments in cultured conditions

**FIGURE 5:**
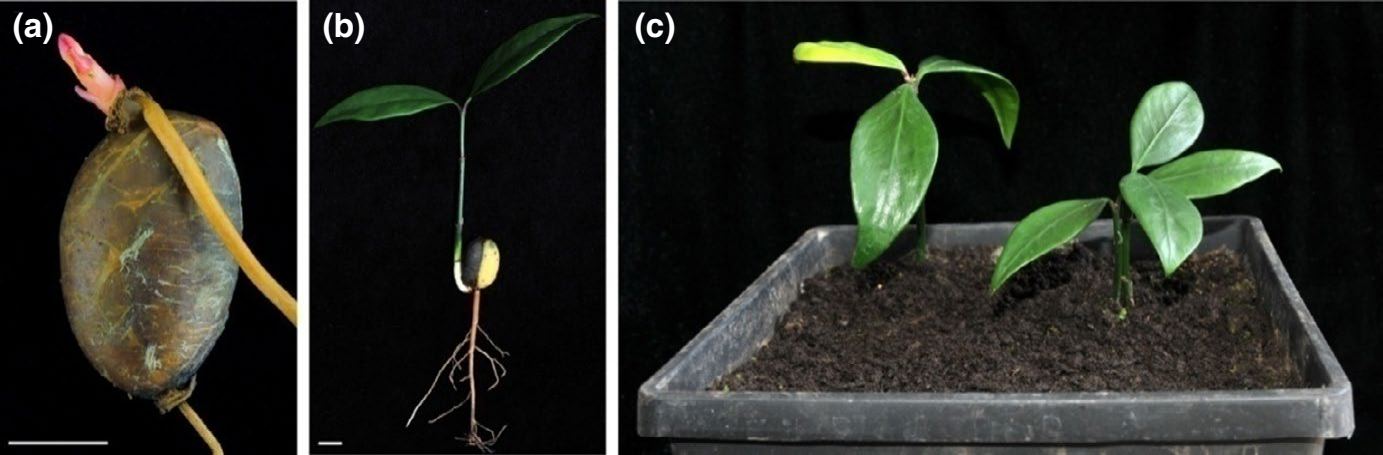
Germination characteristics of intact *G. xanthochymus* seed (a); seedlings of *G. xanthochymus* after planted in the nutrient soil (b, c)

In laboratory conditions, when intact seeds were buried in soil and germinated, they became reddish‐brown, with 2 to 4 pairs of sterile leaves growing up from the seeds (Figure 5a). Seeds did not segregate from seedlings after germination (Figure 5b). Both the young stem and the main root bud sprouted linearly from one end of the cotyledon at the same time, and then, the weaker roots emitted at the other end (Figure 5b). The whole plant could be formed after the germinating seeds were transplanted into nutrient‐rich soil (Figure 5c).

**FIGURE 6:**
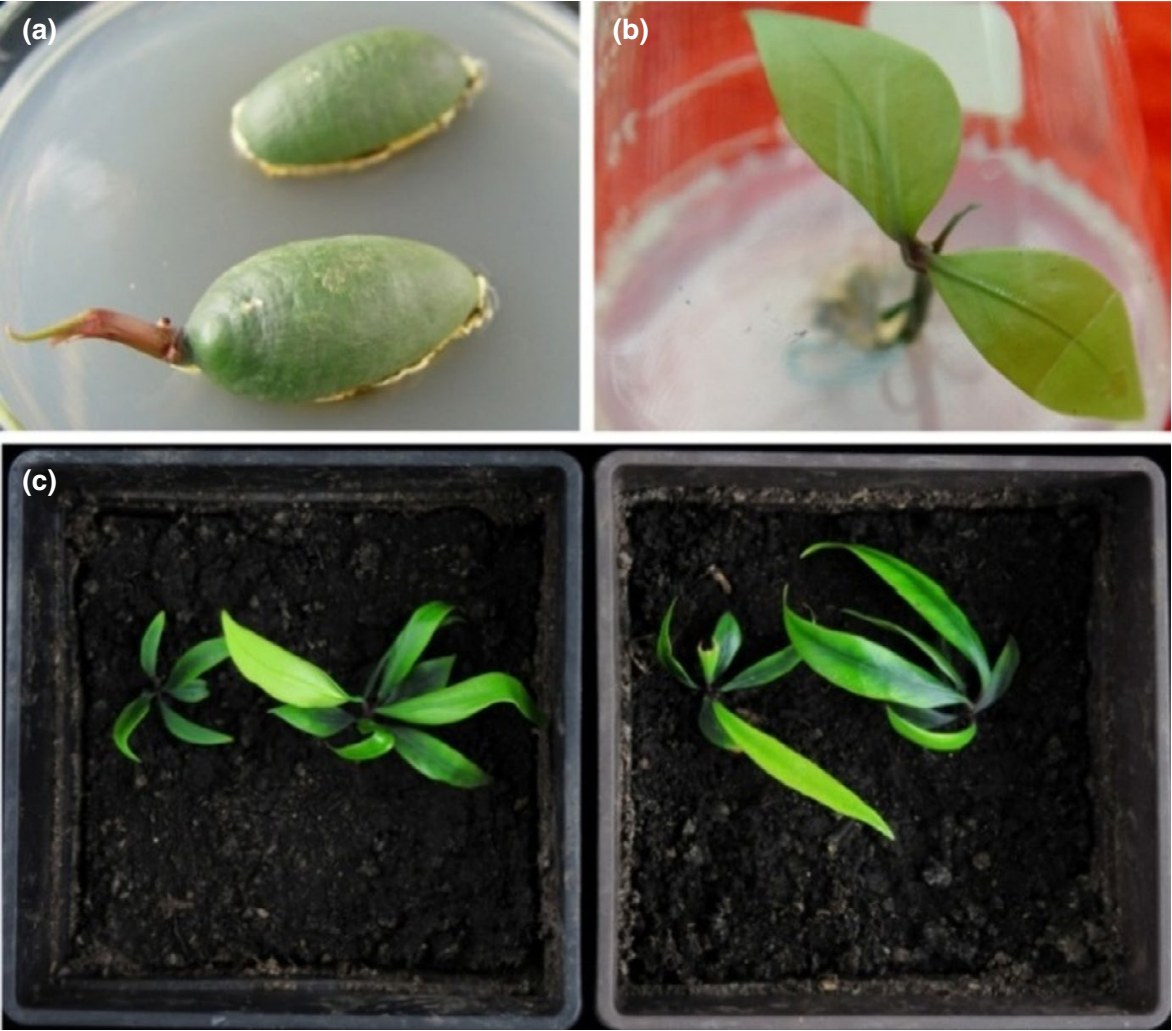
Tissue culture of the *G. xanthochymus* seed in 1/2 MS medium after longitudinal cutting: bud (a) and seedling (b); tissue culture seedlings after transplanting to nutrient soil (c)

Intact seeds were cultured on 1/2 MS medium after longitudinal cutting. Buds were observed in the second week (Figure 6a), and subcultured at about two months, they could form intact seedlings (Figure 6b). The survival rate of these seedlings when transplanted in nutrient‐rich soils exceeded 90% (*n* = 10; Figure 6c).

**FIGURE 7:**
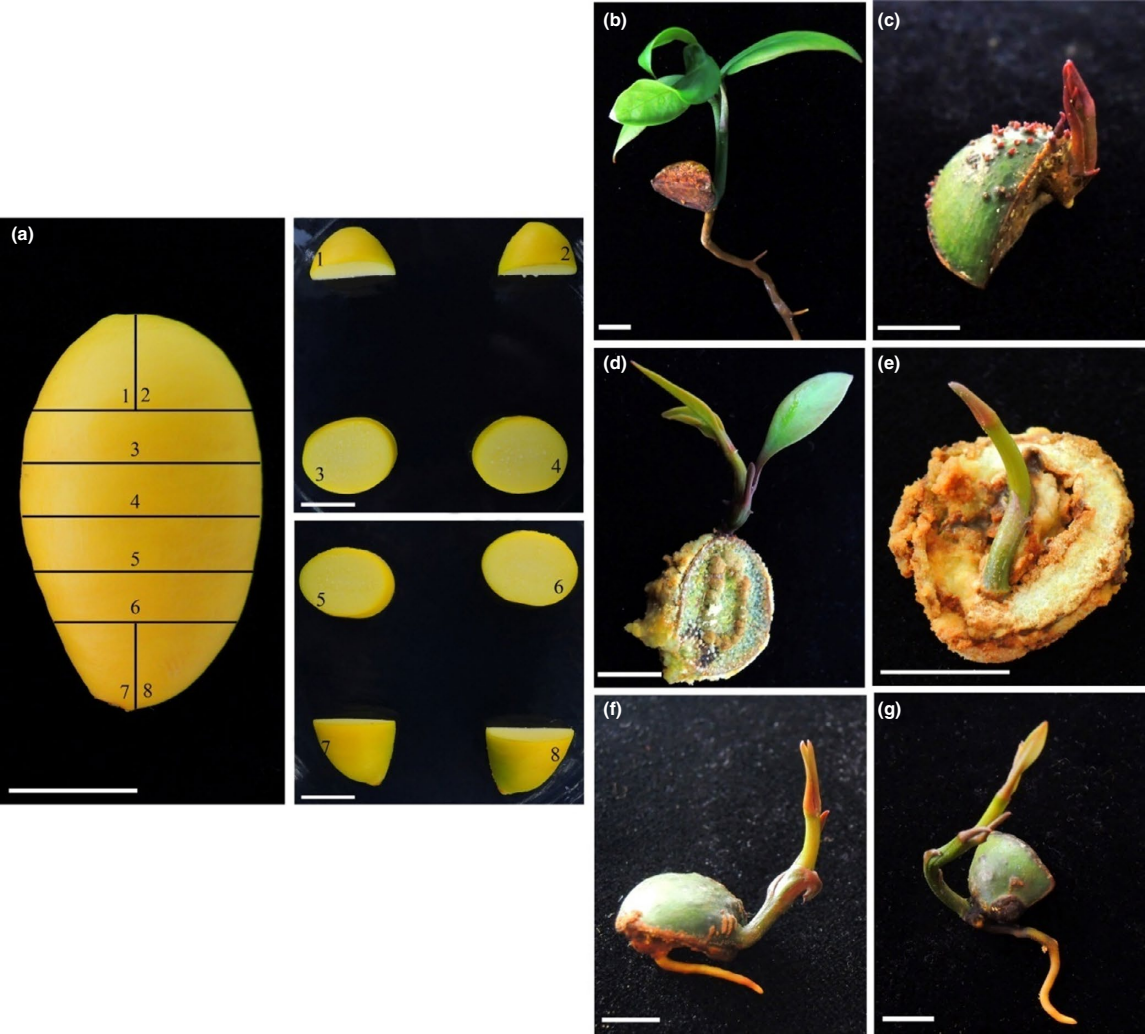
Seed of *G. xanthochymus* used for tissue culture after multiple cutting (a), and germination characteristics of various parts of *G. xanthochymus* seed after multiple cuts: germination from the top of seed (b, c); germination from the middle part of seed (d, e); germination from the base of seed (f, g)

After one *G. xanthochymus* seed was cross‐cut into eight small pieces (see Figure 7a), the pieces were cultured in a 1/2 MS medium supplemented with cytokinin (BA) and auxin (NAA) in matching ratios (Table 1). After one week, the top parts of the seeds cultured in these media were the first to grow small buds (Figure 7b,c). Then, after a subculture, small buds appeared one after another in the middle part of the seed (Figure 7d,e) and at the base (the end with the umbilicus; Figure 7f,g). Many seed fragments cross‐cut from one single seed could establish whole seedlings. The small buds grew faster and produced more seedlings on 1/2 MS medium supplemented with BA and NAA. The other three treatments had little difference in the number of plants produced (Table 1).

**FIGURE 8:**
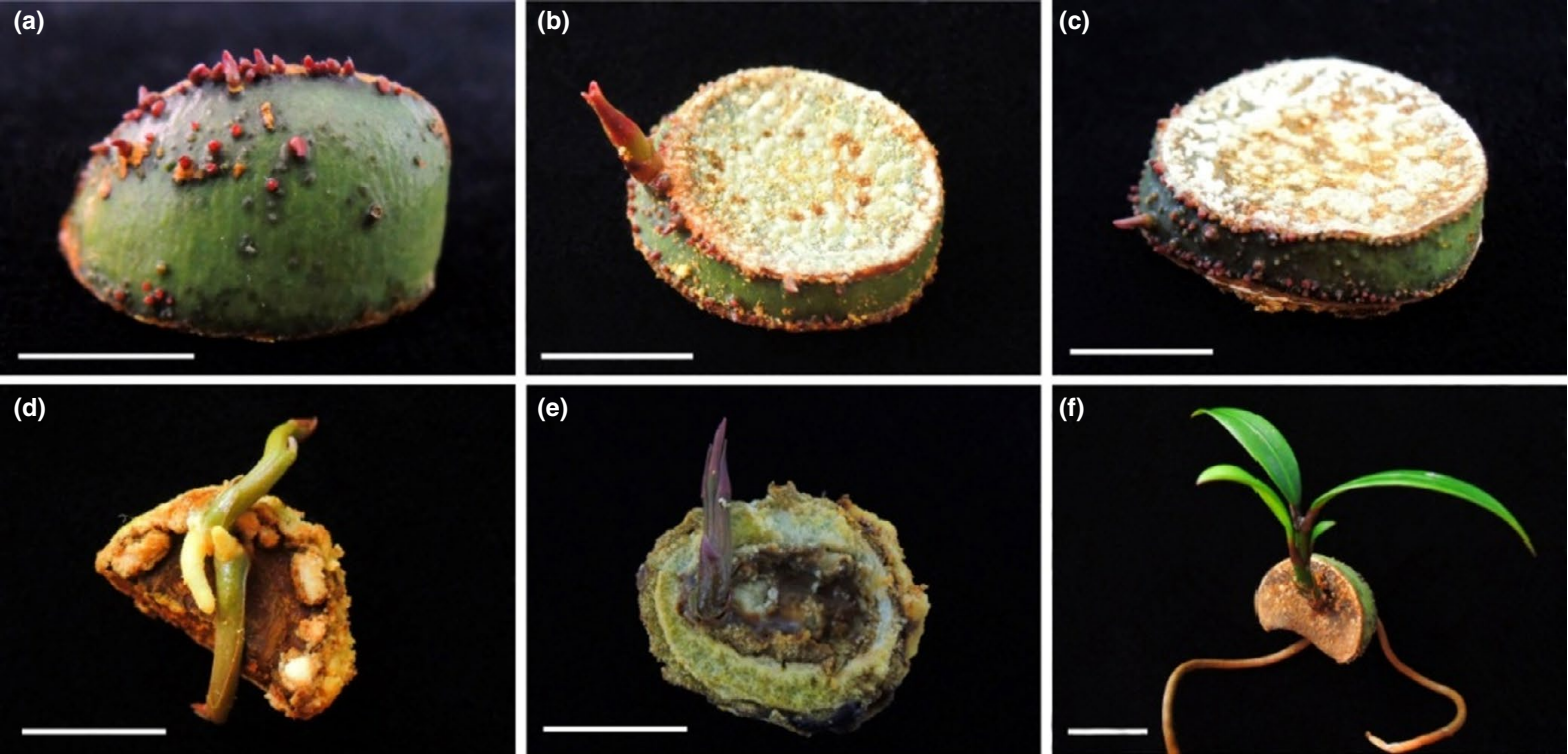
Germination characteristics of various parts of *G. xanthochymus* seed after multiple cuts: buds germinated from the skin of seeds (a, b, c); buds germinated from the internal part of seeds (d, e, f)

During the cultivation process, we found that both the seed epidermis and the inside could germinate small buds and develop into normal plants. Many bud spots could sprout in the seed epidermis (Figure 8a–c), but only a few buds could develop into normal plants (Figure 8b). There are fewer small buds that germinate inside the seed and all will form a complete plant (Figure 8d–f).

## 4 DISCUSSION

In tropical forests of Asia, fruits of Garcinia species produce a sugary water‐rich pulp that larger than 6 cm in diameter which may attract large frugivorous vertebrates for seed dispersal (Anto et al., 2018; Kitamura et al., 2002). However, many large frugivores can destroy seeds during mastication thus impose adverse effect on plant regeneration (Brodie et al., 2017; Campos‐Arceiz et al., 2012). In the Xishuangbanna region, the population of larger frugivorous vertebrates have significantly declined, or even suffered local extinction. Scatter‐hoarding rodents have replaced the roles of primary seed dispersers, playing a significant role in maintaining seedling establishment of *G. xanthochymus*. At the same time, rodents also impose threat to their seeds by predation which often result in many seed fragments as observed in our previous study (Wang et al., 2019). In this study, we found that seeds of *G. xanthochymus* have developed cloning capacity that might help to counter animal predation by reducing the negative outcomes of predation and increasing chances of escape, since both rodent damaged and artificially damaged seed fragments of *G. xanthochymus* were able to germinate and established into normal seedlings, even with up to a 75% loss of their seed mass. Tissue culture experiments further confirmed that all seed fragments contained embryo cells could sprout from both the skin and interior to produce intact seedlings. *G. xanthochymus* seeds have unique properties of having no endosperm and with the embryonic axis extending along the entire seed, which would help seeds to prevent animals from eating all embryo tissues (Figure 1). It is the embryo tissue which has the cloning capacity and not the other seed tissues that came from the parent plant. Our study revealed this novel mechanism of cloning capacity in these plants may be useful in countering animal predation, which likely helps to stabilize the mutualism between plants and animals.

The partial consumption of seeds by animals usually reduces seed vigor and germination success, and it is unfavorable to subsequent growth and development of seedlings (Branco et al., 2002; Jansen et al., 2006; Janzen, 1976; Perea et al., 2011; Xiao et al., 2009). Seeds of many plant species are highly tolerant to animal's partial consumption (Cao et al., 2011; Joshi et al., 2006, 2018; Teixeira & Barbedo, 2012). The germination success and growth performance of damaged seeds largely depend on the structural characteristics of the seeds (such as the morphology and position of embryos or embryonic axis) and the degree of damage to the seeds (Teixeira & Barbedo, 2012; Yi & Yang, 2010; Zhang et al., 2014). Some plant seeds are not able to germinate under more than 10% weight loss (Vallejo‐Marin et al., 2006), while others are able to germinate and successfully develop into normal seedlings with up to 60% of their reserves removed, provided that the vulnerable embryonic parts are not damaged (Giertych & Suszka, 2011; Yi et al., 2015). In our study, we found that intact *G. xanthochymus* seeds germinate easily under natural environmental conditions, and all the germinated seeds established into healthy seedlings. Although artificial cutting (simulating partial consumption by rodents) reduced germination rate and growth of seedlings, the germination rate (83.33%) was remarkably high. Many seeds with up to 75% of their reserves removed successfully geminated and established into healthy seedlings. These findings were consistent with other studies, that both animal natural damaged and artificially damaged seed fragments of seeds of Garcinia species could successfully germinate and establish as seedlings (Anto et al., 2018; Joshi et al., 2006; Malik et al., 2005), implied that the cloning capacity of seed of Garcinia species may be a cope mechanism to animal predation.

The tolerance strategy is widely used by plants to counter animal predation. It emphasizes that the embryo of a seed retains the capacity of establishing into seedling even if it is partially damaged by predators (Dalling & Harms, 1999; Vallejo‐Marin et al., 2006). In contrast to the tolerance strategy, our study indicated that any parts of the *G. xanthochymus* seed could become a seedling, suggesting that seeds of *G. xanthochymus* evolved a cloning capacity to minimize the negative effects of predation. In this study, the cloning strategy was defined here in order to show a different strategy from the tolerance strategy. The cloning strategy emphasizes that the seed is able to produce several seedlings when several seed fragments are produced under predation by the rodents, otherwise only one seedling will emerge.

The cloning capacity of *G. xanthochymus* seeds may be closely related to its unique structural characteristics. Its seed has no endosperm, with the embryonic axis extending along the entire seed. Partial consumption by rodents (or artificially cutting) does not destroy the embryo because all tissues contain embryo cells. This observation was further confirmed in laboratorial tissue culture experiments. Seeds of *G. xanthochymus* were placed in a medium after being cut into many parts, and both the skin and the interior of the seeds could sprout to produce intact seedlings. In our field germination experiments, we found only a few seeds failed to germinate, and some seedlings occasionally died after germination, probably due to infection of saprophytic microorganisms or pathogens or a deficiency in nutrition. It is notable that seed germination rate (38.09%) from the seed fragments of *G. xanthochymus* collected from the enclosures was much lower than that of artificially cut seed fragments (83.33%). There are many reasons for this difference. First is that the rodent damaged seed fragments were temporarily stored in the incubator (10°C) for a few days before the tests, which may have reduced vitality. Second is rodents naturally damage the seeds in a way that prevents seed germination. Third is average mass of seed fragments of *G. xanthochymus* collected from the enclosures was much smaller than artificially cut seed fragments (1.73 ± 0.6 vs. 2.30 ± 0.7). Thus, an additional experiment to evaluate the storage effects, size effects, and differentiate these from the rodent true effect need conduct in future.

For simulated rodent damaged seed fragments, the greater the seed mass retained, the higher the germination probability was, which is consistent with several previous studies (Bonfil, 1998; Mendoza & Dirzo, 2009; Strauss & Agrawal, 1999). In addition, we also found seed mass retained after artificially cutting was significantly and positively correlated with the height of seedlings after germination, while seed mass loss was significantly negatively correlated with the height of seedlings after germination. These results indicated that the nutrients stored in the seeds are not only used for germination, but also used to support the subsequent growth and development of the seedlings (Kennedy et al., 2004; Yi et al., 2015). Seed loss caused by human artificial cutting had an adverse effect on the growth of the seedlings (Mendoza & Dirzo, 2009). The large seed fragments with relatively large reserves grew faster and the seedlings were taller (Kennedy et al., 2004).

Animals not only consume fruits or seeds as seed predators, but also disperse and cache seeds as seed dispersers (Gómez et al., 2019). Thus, tree species and animals form a mutualistic and predatory relationship (Wang et al., 2014). However, over predation of seeds by rodents would shift the mutualism to a predation interaction (Lichti et al., 2017). Antipredation or tolerance strategies are known to alleviate seed predation by animals, which could help to stabilize the mutualism between plants and animals. In this study, we found that in both enclosure and field conditions, rodents eat, and hoard *G. xanthochymus* seeds from the mother tree. Many *G. xanthochymus* seeds were seen to be partially consumed by small rodents, which resulted in seed dispersal of the *G. xanthochymus* (Wang et al., 2019). As compared to intact seeds, these partially eaten seeds were mostly discarded and were rarely eaten or hoarded again by rodents. Because rodent damaged seed fragments of *G. xanthochymus* had cloning capacities, these partially damaged seed fragments were able to grow into seedlings. Indeed, the seedling recruitment rate of tagged seeds released in field was over 20% under rodent predation in field conditions, far higher that most plant species studied in this region (Cao et al., 2011; Wang et al., 2019). Therefore, the cloning capacity of *G. xanthochymus* seeds may help these seeds tolerate predation to a certain extent as an adaptive character ensuring seed dispersal and seedling establishment through a unique plant–animal mutualism. It is necessary to assess the impacts of cloning capacity on final fitness of *G. xanthochymus* seeds and genetic structure of trees in field condition.

## CONFLICT OF INTEREST

The authors have no conflicts of interest to declare.

## AUTHOR CONTRIBUTIONS

**Zhen‐yu Wang:** Data curation (equal); Formal analysis (equal); Investigation (equal). **Lin Cao:** Investigation (equal). **Chuan Yan:** Formal analysis (equal). **Yu‐da Niu:** Methodology (equal). **Kang Chong:** Methodology (equal). **Zhi‐bin Zhang:** Conceptualization (equal); Formal analysis (equal); Funding acquisition (equal); Project administration (equal).

### OPEN RESEARCH BADGES

This article has earned an Open Data Badge for making publicly available the digitally‐shareable data necessary to reproduce the reported results. The data is available at https://doi.org/10.5061/dryad.fn2z34tv0.

## Supporting information

Figure S1

## Data Availability

We archived our data in Dryad (https://doi.org/10.5061/dryad.fn2z34tv0).
