# Supplementary figures and images for: Cloning capacity helps seeds of Garcinia xanthochymus counter animal predation
Source: Ecol Evol. 2021 Aug 23;11(18):12639–50. doi: 10.1002/ece3.8008 (PMC8462166; doi:10.1002/ece3.8008)

**Supporting information**


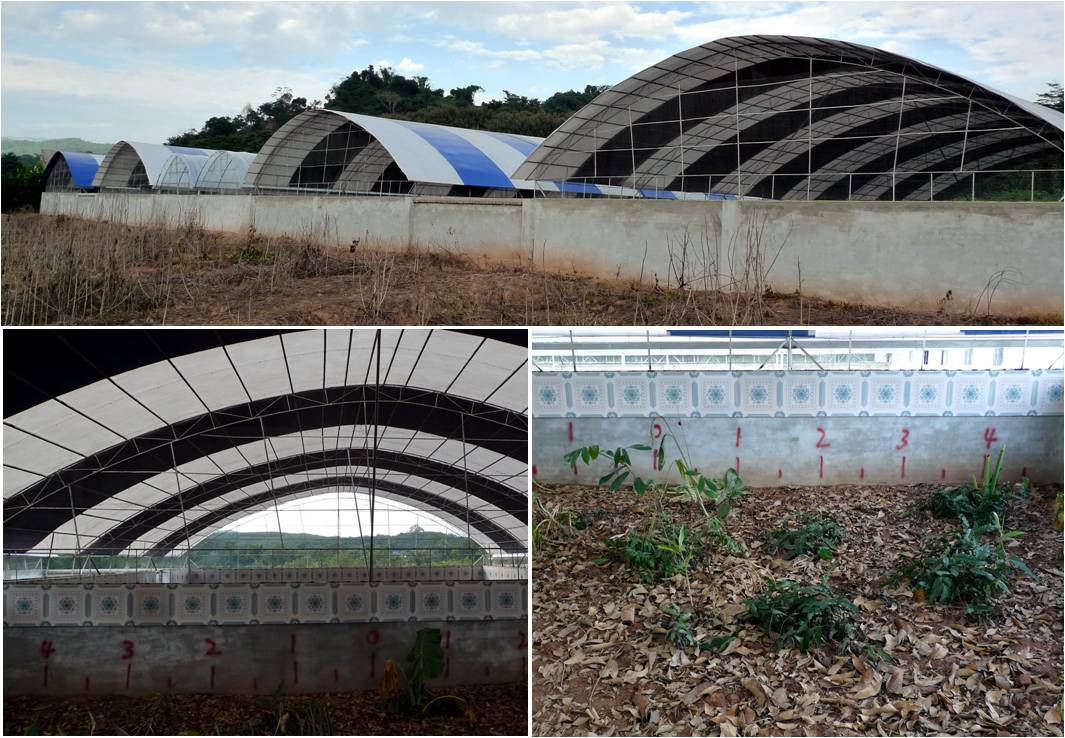


**Fig. S1 The pictures of the semi-natural enclosures.**

Supplement: Supplementary file 1 — Figure S1 [file ECE3-11-12639-s001.docx]
